# Mechanisms underlying thermally induced growth plasticity in juvenile Pacific halibut

**DOI:** 10.1242/jeb.251013

**Published:** 2025-10-06

**Authors:** Josep V. Planas, Andrew J. Jasonowicz, Anna Simeon, Crystal Simchick, Emma Timmins-Schiffman, Brook L. Nunn, Anita C. Kroska, Nathan Wolf, Thomas P. Hurst

**Affiliations:** ^1^ International Pacific Halibut Commission, Seattle, WA 98199, USA; ^2^ Department of Genome Sciences, University of Washington, Seattle, WA 98199, USA; ^3^ FAST Laboratory, Alaska Pacific University, Anchorage, AK 99508, USA; ^4^ Fisheries Behavioral Ecology Program, Alaska Fisheries Science Center, National Marine Fisheries Service, National Oceanic and Atmospheric Administration, Hatfield Marine Science Center, Newport, OR 97365, USA

**Keywords:** Temperature, Skeletal muscle, Fish, Growth biomarkers

## Abstract

Growth plasticity in aquatic ectothermic vertebrates is an important factor driving somatic growth variation in natural populations in response to environmental change. In fish, growth plasticity is primarily due to changes in skeletal muscle growth, as this tissue is a major component of the body mass, with water temperature being a primary abiotic factor affecting growth. Investigating skeletal muscle growth plasticity is therefore key for understanding somatic growth variation. The Pacific halibut (*Hippoglossus stenolepis*) is an important fish species in the North Pacific Ocean ecosystem that has experienced marked changes in size-at-age over the last 100 years. Here, we investigated the molecular basis of growth plasticity in juvenile Pacific halibut acclimated to different temperature regimes under laboratory conditions. By integrating transcriptomic, proteomic and stable isotope analyses of skeletal muscle, we provide evidence for the activation of tiered molecular responses underlying thermally induced growth plasticity. Importantly, we demonstrate that growth plasticity involves plastic molecular responses at the gene, protein and metabolic levels in skeletal muscle that are finely tuned to regulate the synthesis of myofibrillar proteins, among other muscle-related processes. Furthermore, we have identified a set of growth biomarkers that, when tested under field conditions, characterize growth variation among wild individuals. These growth biomarkers, including known and novel growth-related genes, will be useful to elucidate the influence of factors driving somatic growth variation, including changes in size-at-age, in this and other teleost fish species. In summary, this study improves our mechanistic understanding of growth plastic responses to variable temperature regimes in teleost fish and highlights their potential for resilience and/or adaptability in the face of environmental variability.

## Teaser

**Summary:** Integrated transcriptomic, proteomic and stable isotope analyses demonstrate growth plasticity to temperature in juvenile Pacific halibut and identify growth biomarkers that could help characterize somatic growth variation in wild populations.

## INTRODUCTION

Growth plasticity, i.e. the ability of an organism to alter its growth phenotype in response to changes in external factors, is a key feature of the acclimatization process and appears to be universal among all animal species (West-Eberhard, 1989). Among ectothermic fish, water temperature is the primary abiotic factor affecting physiological processes, including growth (Brett, 1979; Jobling, 1996), and most studied species display considerable growth plasticity under thermal variation (Crozier and Hutchings, 2014; Jobling, 1996). Thermal conditions favoring growth leading to increased size can promote fish survival by reducing susceptibility to size-selective predation and by increasing the range of potential prey items (Gibson, 1994). Growth plasticity can therefore be advantageous for individual survival but can also confer resilience to thermal variability under climate change, potentially improving the survival of eurythermal fish species under predicted warmer oceans (Seebacher et al., 2015; Somero, 2010) when energy needed for growth is not restricted.

Growth plasticity is the result of reversible changes in the rate of somatic growth, particularly of growth changes in skeletal muscle as this tissue accounts for more than 60% of the body mass in fish (Bone, 1978; Johnston, 2006). Therefore, understanding skeletal muscle growth is key to explaining somatic growth variation in fish. Skeletal muscle growth in fish results from the accretion of muscle tissue, the great majority of which (>90%) is composed of fast-contracting white muscle fibers (Bone, 1978; Mommsen, 2001). The plastic nature of skeletal muscle growth in fish is acquired ontogenetically during post-embryonic stages once the adult form plan is established, and involves primarily changes in the hypertrophic growth pattern of skeletal muscle fibers (Johnston, 2006). Skeletal muscle growth plasticity is in turn due to plasticity of regulatory processes at the molecular level, namely at the gene, protein and enzyme activity levels. Studies have described plasticity at the level of transcriptional processes in fish skeletal muscle in response to experimental temperature, swimming activity or dietary manipulation (Buckley et al., 2006; Gracey et al., 2004; Palstra et al., 2014; Rescan et al., 2017; Scott and Johnston, 2012). Overall, skeletal muscle growth plasticity has important functional consequences at an organismal level as this tissue provides the engine for swimming because of its contractile nature, therefore influencing movement and migratory capabilities, and plays an important role in whole-body metabolic homeostasis (Guderley, 2004; Mommsen, 2001).

At the population level, growth plasticity in fish has a strong influence on population biomass variation over temporal and spatial scales (Lorenzen, 2016; Stawitz and Essington, 2019). A likely manifestation of growth plasticity is the change in average size-at-age observed in several exploited fish populations (Lorenzen, 2016). The Pacific halibut (*Hippoglossus stenolepis*) is one of the largest flatfish species in the North Pacific Ocean, reaching over 200 kg in mass and 2.4 m in length, and provides an excellent example of an ecologically, culturally and commercially important migratory fish species with a long historical record of decadal changes in average size-at-age (Clark et al., 1999; Clark and Hare, 2002). Pacific halibut size-at-age steadily increased from the 1920s until historical highs in the 1990s and subsequently declined to levels comparable to those at the beginning of the time series (Clark and Hare, 2002). It is estimated that the average mass of an adult female Pacific halibut decreased ∼50% between the late 1970s and the early 2000s, resulting in important changes in population biomass (Clark and Hare, 2002). The reasons for this change in size-at-age in Pacific halibut are still not well understood despite past efforts to relate it to environmental conditions (e.g. temperature), ecological factors (e.g. intra- and inter-specific competition, prey quality or availability) and fisheries-induced effects (e.g. size-selective removal of larger individuals) (Barnes et al., 2018; Clark and Hare, 2002; Clark et al., 1999; Hilborn and Minte-Vera, 2008; Holsman et al., 2018; Sullivan, 2016; Wolf et al., 2019). Regardless of which of these factors individually or in combination affect size-at-age variation in Pacific halibut, these are ultimately the result of variation in the growth process leading to that state (Enberg et al., 2012). Laboratory studies investigating the effects of temperature on growth in juvenile Pacific halibut at an individual level have confirmed the thermal influence on growth and the capacity for compensatory growth (Hurst et al., 2005). These studies indicate that environmental temperature variation can cause plastic growth responses in juvenile Pacific halibut that are typically found in relatively shallow benthic habitats (<40 m in depth at age 0) (Norcross et al., 1995, 1999) where variation in local conditions may have a considerable effect on juvenile growth rates.

However, the molecular mechanisms underlying growth responses to temperature in Pacific halibut have not been examined to date. The present study represents the first attempt at investigating the molecular basis of growth changes in juvenile Pacific halibut in response to acclimation to different temperature regimes under experimental conditions. To address this question, we have taken a systems-biology approach to investigate growth processes at the gene, protein and metabolic levels in white skeletal muscle, providing insights into tiered molecular responses to thermally induced growth changes. By combining transcriptomic and proteomic analyses, we have identified a large collection of expressed genes and proteins in skeletal muscle that participate in key somatic growth processes supported by stable isotopic data. The identified molecular signatures of growth in skeletal muscle can potentially inform population-level growth patterns in this important species.

## MATERIALS AND METHODS

### Ethics statement

Ethical review and approval was not required for the animal study because biological samples were collected as part of survey activities conducted under a letter of acknowledgment from the National Oceanographic and Atmospheric Administration (NOAA) Fisheries division. Nonetheless, biological samples were collected under the guidelines for the euthanasia of finfish as outlined by the American Veterinary Medical Association (AVMA, 2020).

### Fish collection and holding for laboratory experiments

**Fig. 1.**
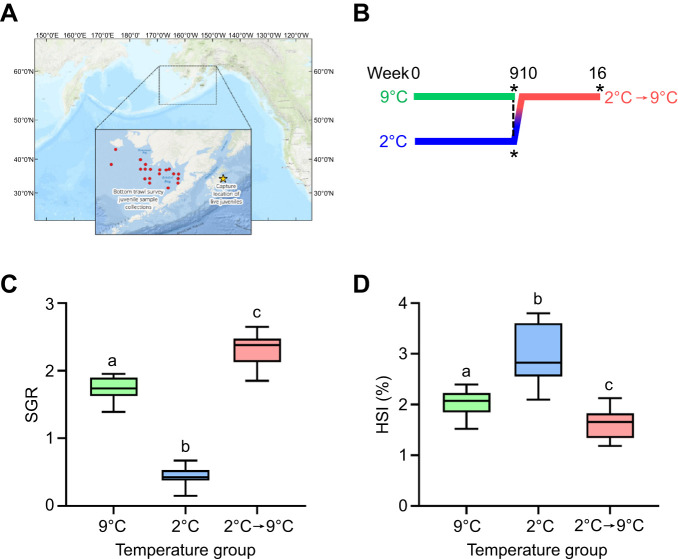
**Experimental protocol and temperature acclimation effects on specific growth rate and hepatosomatic index.** (A) Map of the North Pacific Ocean area showing the capture location of live Pacific halibut juveniles (star) off Kodiak Island, and the sampling locations of juvenile Pacific halibut on the NMFS-NOAA bottom trawl survey (red dots) in the Eastern Bering Sea shelf. (B) Graphical representation of the experimental protocol. The dashed line indicates the beginning of the transition from 2°C to 9°C at week 9, and asterisks indicate the time points when fish sampling occurred. (C) Effects of temperature acclimation on specific growth rate (SGR). (D) Effects of temperature acclimation on hepatosomatic index (HSI). Box plots show the median, first and third quartiles, and minimum–maximum range (*N*=10). Outliers are shown by individual points and were not excluded from statistical analyses. Statistically significant (*P*<0.01, Tukey's *post hoc*) differences among groups are indicated by different letters.

Juvenile (age 0) Pacific halibut (*Hippoglossus stenolepis* Schmidt 1904) were collected in July 2016 from Gulf of Alaska nearshore waters around Kodiak Island, AK, USA (Fig. 1A) with a 2 m beam trawl in multiple tows conducted over several days at depths of 5–30 m, with average temperature and salinity of 12°C and 31.4‰, respectively. After capture, fish were transferred to tanks with flow-through ambient seawater at the Alaska Fisheries Science Center (AFSC) laboratory in Kodiak. After several days of holding, fish were shipped overnight in insulated containers to the AFSC laboratory in Newport, OR, USA.

Prior to use in experiments, fish were acclimated in 1 m diameter tanks at 7–9°C, fed thawed krill and a gelatinized combination of krill, squid, herring and nutritional supplements to satiation daily for approximately 1 month, and subsequently fed 3 times per week. Fish were individually marked with passive integrated transponder (PIT) tags (Biomark MiniHPT8; Boise, ID, USA) inserted into the body cavity. Fish resumed feeding within 48 h of tagging and there were no mortalities associated with the tagging procedure. Fish were allowed to recover from tagging for 2 weeks prior to being moved to the experimental tanks.

### Temperature-induced growth manipulation laboratory experiments

The experimental design to characterize temperature effects and compensatory growth responses in juvenile Pacific halibut largely followed a previously described design (Hurst et al., 2005). Five fish were randomly assigned to each of six experimental tanks (30 fish total; 64.90±8.98 mm tail length, 2.81±1.21 g wet mass; means±s.d.). Tanks were 46×64 cm with 12 h daily illumination provided by overhead fluorescent fixtures and received a continuous flow of temperature-controlled seawater at 9°C. Following 1 week of acclimation, the temperature of four of the tanks was lowered to 2°C by mixing ambient and chilled seawater over the course of 4 days (<2°C day^−1^); the temperature in the remaining two tanks was maintained at 9°C. Fish were held at these temperatures for an additional 2 weeks of acclimation and fed to satiation 3 times per week prior to initiating growth measurements. Following this acclimation period (and 24 h following prior feeding), all fish were captured from their tanks, identified by PIT scanning, weighed (to 0.01 g), measured (to 1.0 mm) and returned to their tanks. During the experimental phase, fish were fed to satiation daily, except on the day prior to measuring. All fish were measured after 2, 4, 6, 8 and 9 weeks from the start of the experiment. At the 9-week sampling point, fish from the 9°C tanks and two of the four 2°C tanks were killed with an overdose of anesthetic (MS-222; Sigma-Aldrich, St Louis, MO, USA) for biological sampling. The temperature in the remaining two 2°C tanks was gradually increased to 9°C during a 7 day period (1°C day^−1^) to elicit a compensatory growth response (treatment referred to as 2°C→9°C). Following 14 days of acclimation at 9°C, fish were weighed and measured at 2 week intervals (total of 12, 14 and 16 weeks from the start of the experiment). After the 16 week sampling time point, the remaining fish were killed for biological sampling, as described above. A graphical representation of the experimental protocol can be found in Fig. 1B.

On the final sampling day for each treatment, fish were captured from the rearing tank, weighed, measured and killed. Livers were removed and weighed to calculate the hepatosomatic index (HSI, liver mass/body mass×100) as an indicator of condition. Samples (∼100–500 mg) of dorsal white skeletal muscle tissue were dissected from the ‘eyed side’ and either frozen or preserved in RNAlater (Invitrogen, Carlsbad, CA, USA) following the manufacturer's specifications, and stored at −80°C until processed for analysis. Growth rates of each fish in the experiment were calculated using the measurements made throughout the experiment (excluding temperature acclimation periods). Growth rates in length (mm day^−1^) and mass (specific growth rate, SGR) were determined by regressing fish length and natural log-transformed mass, respectively, against sampling date (Hurst et al., 2005). For the fish that were reared at 2°C before being increased up to 9°C, growth rate was calculated separately for each rearing phase. In order to account for possible size-based differences in underlying growth potential, growth rates of fish in the 9°C treatment were also calculated over weeks 4–9 (corresponding to the body sizes of thermally delayed fish measured during weeks 12–16; Hurst et al., 2005).

### Collection of white muscle samples from wild juveniles

To evaluate the robustness of patterns of gene expression observed in the laboratory experiment, wild juvenile Pacific halibut were captured during the National Marine Fisheries Service – National Oceanic and Atmospheric Administration summer bottom trawl survey in the Eastern Bering Sea shelf (Fig. 1A). Fork length (FL) was measured, right sagittal otoliths were collected for surface aging, and dorsal white muscle tissue samples were excised, preserved in RNAlater and stored as described above. Once fish were aged, we searched for age classes containing at least 10 individuals in each of three size categories (small: 30–40 cm FL; medium: 40–50 cm FL; large: 50–60 cm FL) and only juveniles at 4 years of age fitted those criteria. Therefore, skeletal muscle samples from 4-year-old fish were chosen for evaluating potential differences in gene expression among age-matched juveniles of different size categories.

### Transcriptome analyses by RNA sequencing

White muscle samples in RNAlater from the three experimental groups (2°C, *n*=8; 9°C, *n*=8; 2°C→9°C, *n*=8) were processed for total RNA extraction, library preparation and sequencing by Omega Bioservices (Norcross, GA, USA). All samples passed the quality control, with RNA integrity numbers ranging between 9.1 and 10. Samples were sequenced using the next generation sequencing (NGS) platform Illumina HiSeq2500 in read format paired-end 2×100 bp with a coverage per sample of 30 million reads. Data filtering (using Trimmomatic v0.30), read alignment against the reference zebrafish genome GRCz10 (using Tophat; 88.8% overall read alignment rate), transcript assembly and annotation and transcript differential expression (using Cufflinks) were performed by the Omega Bioservices bioinformatics pipeline. Sequence enrichment analyses of transcriptomic data were performed using OmicsBox (v1.2) (Gotz et al., 2008). Briefly, a two-tailed Fisher's exact test was used to detect over- and under-represented gene ontology (GO) terms associated with the differentially expressed genes (DEGs) for each temperature treatment. The RNA sequencing (RNA-seq) data are openly available from NCBI under BioProject PRJNA635611. To visualize common and specific DEGs associated with temperature-induced growth manipulations, Venn diagrams were constructed using the matplotlib-venn (v0.11.6, https://github.com/konstantint/matplotlib-venn) package in python (v3.8, https://www.python.org) from lists of DEGs filtered to select for genes that were significantly up-regulated during growth stimulation (9°C versus 2°C→9°C) and significantly down-regulated during growth suppression (9°C versus 2°C). Shared DEGs between these two lists were identified as candidate genes associated with temperature-induced growth manipulations.

### RT-qPCR

To validate the results obtained from the transcriptomic analysis, RT-qPCR was performed on RNA samples from the temperature-induced growth manipulation experiments for selected DEGs. In addition, expression of the same set of genes was measured by RT-qPCR using RNA from wild juvenile muscle samples extracted using Trizol (Invitrogen). Total RNA (1 μg) was treated with DNAse I Amplification Grade (Invitrogen) to remove any contaminating genomic DNA. Resulting RNA concentrations were measured on a NanoDrop 8000 spectrophotometer (Life Technologies, Carlsbad, CA, USA), and 20 ng of each RNA sample was reverse transcribed using the SuperScript IV VILO Master Mix cDNA kit (Invitrogen) using the protocol specified by the manufacturer. All PCR reactions were run in a QuantStudio 6 Real-Time PCR System (Life Technologies) and followed the requirements of the MIQE guidelines (Bustin et al., 2009). Each 10 µl reaction was run in triplicate and contained 2 µl of cDNA (diluted 1:30), 400 nmol l^−1^ of each primer and PowerUp SYBR Green Mastermix (Life Technologies) using the manufacturer's standard PCR and melt curve protocols. The specificity of the reaction and absence of primer dimers were confirmed by the melting curve profile, and products of new primer pairs were run on a 1.5% agarose gel to confirm expected amplicon size. Primer sequences were designed in Primer3 (Untergasser et al., 2012) using Geneious (Table S1) and efficiency was calculated by analyzing serial dilutions of cDNA samples. Potential housekeeping genes were analyzed using geNorm and NormFinder which identified glyceraldehyde 3-phosphate dehydrogenase (*gapdh*) and ubiquitin (*ubq*) as the most suitable. The expression level of each target gene was normalized to that of those two reference genes (*M*-value <0.5) and calculated using the 2^−ΔΔ*C*_T_^ method (Livak and Schmittgen, 2001).

### Proteome analyses

#### Protein digestion, desalting and liquid chromatography–tandem mass spectrometry (LC-MS/MS)

Pacific halibut white muscle samples from the three experimental groups (*n*=10 per group) were homogenized in 500 μl of 50 mmol l^−1^ NH_4_HCO_3_ with 6 mol l^−1^ urea. The muscle homogenate was subjected to trypsin digestion and desalting as previously described (Timmins-Schiffman et al., 2013) with additional digestion using lysyl endopeptidase enzyme (LysC; Wako Chemicals, Richmond, VA, USA) (Maas et al., 2018). Dried desalted peptides were reconstituted in 100 µl 5% acetonitrile (ACN) with 0.1% formic acid. Digested peptides were analyzed on a Q-Exactive mass spectrometer (Thermo Scientific, Waltham, MA, USA) using data-dependent acquisition. Chromatography separation of peptides before MS/MS analysis was done with an Easy-LC (Thermo) system with a 100 µm pre-column (4 cm) and 75 µm analytical column (30 cm) with a laser-pulled tip to 5 µm, both packed in-house with 3 µm C18 beads (Dr Maisch HPLC GmbH, Ammerbuch, Germany). Samples were analyzed in a randomized order with 2 µg of Pacific halibut peptides injected in two distinct instances. Peptides were eluted off the columns over 240 min with a gradient of 5–45% of 80% ACN. In MS1 analysis in the Orbitrap, the resolution was 70,000, scan range was 375–1575 *m*/*z*, maximum injection time was 100 ms, loop count was 20, scan range was 200–2000 *m*/*z* and AGC target was 1e6. During MS2 analysis, the resolution was 17,500, maximum injection time was 50 ms and AGC target was 1e5, and centroid data were collected. The mass spectrometry data have been deposited in the ProteomeXchange Consortium via the PRIDE (Perez-Riverol et al., 2019; Vizcaíno et al., 2016) partner repository with the dataset identifier PXD012634.

#### Protein inference

Acquired peptide spectra were searched against a transcriptome-derived Pacific halibut muscle database. RNA from red and white muscle tissue was previously sequenced as described above, and data are available in NCBI under BioProject PRJNA634339. The nucleotide fasta files were analyzed with Transdecoder within the Trinity pipeline to derive predicted protein sequences, resulting in 27,682 and 76,873 predicted sequences from white and red muscle, respectively. These databases were combined and sequence redundancy was removed using cd-hit (Li et al., 2001) for a final database of 52,639 protein sequences. Protein sequences from common laboratory contaminants were added to the Pacific halibut muscle protein database (CRAPome; Mellacheruvu et al., 2013) and the final databases can be found at PRIDE repository PXD012634. Spectra were searched against the Pacific halibut muscle protein database using Comet v2018.01 rev.3 (Eng et al., 2013, 2015) followed by the Trans-Proteomic Pipeline (TPP; Keller et al., 2002; Nesvizhskii et al., 2003) to derive the probability of each peptide and protein inference. TPP was run with a probability cut-off of 0 as the final false discovery rate (FDR) was determined from a combined prot.xml file in the Abacus (Fermin et al., 2011) pipeline. Abacus was used to find consensus protein inferences across all biological and technical replicates with an FDR cut-off of 0.01 (probability of 0.9). The adjusted normalized spectral abundance factor (NSAF) calculated by Abacus was used as a proxy for relative protein abundance.

#### Proteomic data analysis

Technical replication across duplicate sample injections was consistent and, consequently, protein abundance values were averaged across technical replicates for each sample. NSAF values were log(*x*+1)-transformed and non-metric multidimensional scaling (NMDS), analysis of similarity (ANOSIM) and identification of differentially abundant proteins (DAPs) was performed as previously described (Maas et al., 2018; Timmins-Schiffman et al., 2013). Pacific halibut protein sequences (*n*=3140) were annotated with the trembl database using blastp (Camacho et al., 2009) with an e-value cut-off of 1e^−10^. The database was downloaded from UniProt in July 2018. Enrichment analysis was used to reveal functional categories of proteins that are over-represented in a dataset compared with a background (usually the entire sequenced proteome). Groups of proteins that were detected at significantly higher or lower levels in pairwise temperature comparisons in QSpec were used as input protein lists in the enrichment analysis with all detected proteins in the proteome as a background. The in-house enrichment tool compGO (Timmins-Schiffman et al., 2017), for this project can be accessed via https://meta.yeastrc.org/compgo_halibut/pages/goAnalysisForm.jsp. GO terms for each group of proteins were considered significantly enriched compared with the background proteome at a *P*-value cut-off of 1e^−10^. The same analysis was performed on the three groups of proteins identified in each temperature treatment.

### Transcriptomic and proteomic comparisons

A comparison between DEGs and DAPs was made to identify parallel responses at both the transcriptomic and proteomic levels. We first mapped the UniProt AC/ID to the UniProt Reference Clusters database (UniRef50% UniRef90) using the UniProt ID mapping tool (https://www.uniprot.org/id-mapping/). This enabled comparisons of the DEG and DAP lists to be made at varying levels of sequence similarity and allowed transcripts and proteins that may have similar but not exact UniProt AC/ID annotations to be grouped together. We then searched the lists for any UniRef IDs that were shared between the DEG and DAP lists.

### Stable isotope analyses

Pacific halibut white muscle samples were oven-dried to a constant mass at 42°C. Each sample was ground to a fine powder and approximately 0.5–1.0 mg was loaded into individual tin capsules for stable isotope analysis. δ^13^C and δ^15^N values of the tissue samples were determined using a Carlo Erba 1110 Elemental Analyzer (Carlo Erba Reagents, CE Instruments, ThermoQuest Italia S.p.A., Milan, Italy) coupled to a Thermo Delta Plus XP IRMS (Thermo Finnigan, Bremen, Germany) at the University of Wyoming's Stable Isotope Facility (Laramie, WY, USA). Long-term analyses of quality control standards have yielded precisions of 0.3‰ for δ^13^C and 0.4‰ for δ^15^N. Stable isotope data are presented in ‰ relative to Pee Dee Belemnite for ^13^C and atmospheric nitrogen for ^15^N.

Isotopic profiles for each temperature treatment group were assessed using Bayesian implementations of standard ellipse areas and centroid locations calculated using the Stable Isotope Bayesian Ellipses in R package (SIBER; Jackson et al., 2011) in R. Standard ellipses were created using Bayesian estimates with the default SIBER settings, where Markov chain Monte Carlo length=1,000,000, burn in=1000, thinning=10, chains=2 and 40% of the data is included in ellipse construction. Overlap among ellipses was assessed using Bayesian estimates of overlap expressed as proportions of the non-overlapping areas of the ellipses. Differences in the locations of ellipse centroids among temperature treatment groups were assessed using pairwise comparisons of polar vectors. Trophic discrimination values (Δ^13^C or Δ^15^N) were calculated as δ*^X^X*_tissue_−δ*^X^X*_diet_. Differences in mean trophic discrimination values among treatment groups were explored using one-way analysis of variance (ANOVA).

### Statistical analyses

Statistical differences among mean SGR and HSI values of the three experimental groups were analyzed by ANOVA followed by Tukey's multiple comparisons test and unpaired *t*-test. Statistical differences among mean mRNA expression levels, expressed as fold-change relative to either the 9°C group or to the small size group, were analyzed by the non-parametric Kruskal–Wallis test followed by Dunn's multiple comparisons test. SGR, HSI and gene expression results are shown as means±s.e.m. and were considered to be significant at *P*<0.05. All statistical analyses were performed using GraphPad Prism 9.5.1.

## RESULTS

### Temperature-induced growth manipulations

Experimental temperature manipulations resulted in significant changes (ANOVA, d.f.=2, *P*<0.0001) in SGR in juvenile Pacific halibut (Fig. 1C). Low-temperature (2°C) acclimation for 9 weeks resulted in a ∼75% decrease (*P*<0.0001) in SGR relative to fish acclimated at 9°C. In contrast, fish that were acclimated to 2°C for 9 weeks and were subsequently acclimated to 9°C for an additional period of 7 weeks (2°C→9°C) experienced a ∼25% increase (*P*<0.0001) in SGR relative to fish held continuously at 9°C. Final average mass (means±s.e.m.) differed among the three temperature groups: 7.91±1.41, 3.34±0.32 and 8.53±1.07 g for fish from the 2°C, 9°C and 2°C→9°C groups, respectively. HSI was higher (*P*<0.0001) in fish held at 2°C than in fish acclimated at 9°C but was lower (*P*<0.01) in fish from the 2°C→9°C treatment than in fish held continuously at 9°C (Fig. 1D).

### White muscle transcriptomic profiling of temperature-induced growth manipulations

**Fig. 2.**
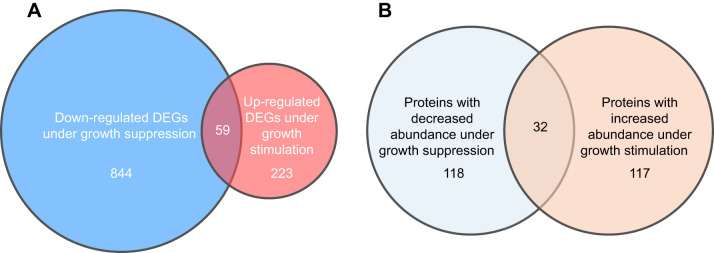
**Venn diagrams of differentially expressed genes (DEGs) and differentially abundant proteins (DAPs) of white muscle that are specific for each temperature-induced growth manipulation, and common DEGs and DAPs.** (A) DEGs; (B) DAPs. Numbers indicate the number of specific and common DEGs and DAPs.

We conducted transcriptomic profiling of white muscle of juvenile Pacific halibut by RNA-seq to identify genes that change their expression levels in the same direction as temperature-induced growth manipulations. First, we compared white muscle samples from fish subjected to low temperature-induced growth suppression (i.e. fish acclimated to 2°C) and control fish (i.e. fish held continuously at 9°C) and identified 1619 DEGs, of which 1187 were annotated (Table S2). Among all DEGs, 716 (of which 511 were annotated) showed increased mRNA expression levels (i.e. up-regulated) in fish acclimated at 2°C when compared with fish held at 9°C. In contrast, 903 DEGs (of which 676 were annotated) showed decreased mRNA expression levels (i.e. down-regulated) in fish acclimated at 2°C when compared with fish held at 9°C (Fig. 2A; Tables S2 and S3). Enrichment analyses of DEGs with decreased expression in fish subjected to growth suppression evidenced significant over-representation of biological process GO terms related mostly to immune response, muscle contraction and muscle system process, comprising a number of down-regulated DEGs encoding proteins with actin binding function that are constituents as well as functional regulators of muscle contractile fibers (Table S4). Top down-regulated DEGs encode proteins known to participate in muscle contraction (e.g. *acta1*, *cftr*, *myh7*, *myss*, *ryr1*, *tnnt3*), muscle development (e.g. *flnc*, *obscn*, *peg3*, *rbbp7*), actin cytoskeleton and filament organization (e.g. *coro1a*, *pip5k1c*), protein synthesis and translation (e.g. *asns*, *ass1*, *eef1a1*, *gars*, *rpl35*), glucose metabolism (*tigara*) and the immune response (*lysc1*) (Table S3). (Full gene names are given in Table 1 and Tables S3 and S5. Gene nomenclature according to https://zfin.org.)

**Table 1.** List of common differentially expressed genes (DEGs) in white muscle that are down-regulated in response to growth suppression by low-temperature acclimation (9°C vs 2°C) and up-regulated in response to growth stimulation by temperature-induced compensatory growth (9°C vs 2°C→9°C)

UniProt ID	UniProt name	UniProt description	Gene name
P53480	ACTC_TAKRU	Actin, alpha cardiac	*LOC101062076*
P68264	ACTS_OREMO	Actin, alpha skeletal muscle	*acta1*
P08023	ACTA_CHICK	Actin, aortic smooth muscle	*acta2*
Q7T3R4	RHCG2_TAKRU	Ammonium transporter Rh type C 2	*rhcg2*
Q5ZJU3	ASNS_CHICK	Asparagine synthetase [glutamine-hydrolyzing]	*asns*
Q9UKI2	BORG2_HUMAN	Cdc42 effector protein 3	*cdc42ep3*
Q6IQM2	CYC_DANRE	Cytochrome *c*	*cyc*
Q6AZ83	G6PC3_RAT	Glucose-6-phosphatase 3	*g6pc3*
Q5SPB6	CHAC1_DANRE	Glutathione-specific gamma-glutamylcyclotransferase 1	*chac1*
Q5I0G4	SYG_RAT	Glycine--tRNA ligase	*gars*
Q9PVM4	HBAA_SERQU	Hemoglobin subunit alpha-A	*hbaa*
Q9PVM2	HBBA_SERQU	Hemoglobin subunit beta-A	*hbb1*
Q8N7A1	KLDC1_HUMAN	Kelch domain-containing protein 1	*klhdc1*
Q05AX4	MSTN1_XENLA	Musculoskeletal embryonic nuclear protein 1	*mustn1*
Q90339	MYSS_CYPCA	Myosin heavy chain, fast skeletal muscle	*myss*
P97434	MPRIP_MOUSE	Myosin phosphatase Rho-interacting protein	*mprip*
P02609	MLRS_CHICK	Myosin regulatory light chain 2, skeletal muscle isoform	*mylpf*
Q9Y2K3	MYH15_HUMAN	Myosin-15	*myh15*
P00480	OTC_HUMAN	Ornithine carbamoyltransferase, mitochondrial	*otc*
Q9H4L5	OSBL3_HUMAN	Oxysterol-binding protein-related protein 3	*osbpl3*
Q3SYZ8	PDLI3_BOVIN	PDZ and LIM domain protein 3	*pdli3*
Q29RA5	TIGRA_DANRE	Probable fructose-2,6-bisphosphatase	*tigara*
Q4QR77	F166A_RAT	Protein FAM166A	*fam166a*
Q4VC12	MSS51_HUMAN	Putative protein MSS51 homolog, mitochondrial	*mss51*
Q8WZ42	TITIN_HUMAN	Titin	*ttn*
P02645	TNNI1_RABIT	Troponin I, slow skeletal muscle	*tnni1*

Gene nomenclature according to https://zfin.org.

Second, we compared white muscle samples from fish subjected to temperature-induced growth stimulation (2°C→9°C treatment) and control fish (i.e. fish held continuously at 9°C), and identified 815 DEGs, of which 610 were annotated (Table S2). Among all DEGs, 282 were up-regulated (202 annotated) in fish experiencing growth stimulation (Fig. 2A; Tables S2 and S5). In contrast, 533 DEGs were down-regulated (408 annotated) in fish experiencing growth stimulation (Table S2). A closer examination of DEGs with increased expression in fish experiencing growth stimulation showed significantly enriched biological process GO terms related to oxygen transport, immune response, lipid metabolism, RNA metabolism, gene expression and actin-mediated cell contraction and movement, the latter in the context of muscle contractile fibers as shown by the corresponding cellular component GO terms (Table S6). Top up-regulated DEGs encode proteins known to participate in hemoglobin synthesis and hemoglobin-mediated oxygen transport (e.g. *eif2ak1*, *hba*, *hbb*), muscle contraction and development (e.g. *ata1*, *il7r*, *lrrc2*, *myh2*, *mgp*, *neu3*, *obscn*, *ttn*, *usp25*), and glucose and glycogen metabolism (e.g. *pgk1*, *tigara*, *trim7*, *vdac1*) (Table S5).

To identify genes that could represent potential molecular markers of temperature-regulated growth in white muscle of juvenile Pacific halibut, we set out to identify DEGs that showed changes in expression levels that paralleled observed changes in SGR in the two types of temperature-induced manipulations. Specifically, we compared DEGs that were down-regulated under growth suppression with DEGs that were up-regulated under growth stimulation and identified 59 common DEGs, of which 26 were annotated (Fig. 2A; Table 1). Among these, the great majority of common DEGs encode proteins that participate in key skeletal muscle processes, including protein synthesis (e.g. *asns*, *gars*), sarcomeric structure and function (e.g. *acta1*, *myh15*, *mylpf*, *myss*, *tnni1*, *ttn*), muscle development and regeneration (e.g. *chac1*, *mustn1*) and actin cytoskeleton (e.g. *cdc42ep3*, *mprip*, *osbpl3*). Common DEGs also encode proteins involved in the mitochondrial respiratory chain (e.g. *cyc*, *mss51*), oxygen transport (e.g. *hba*, *hbb*) and carbohydrate metabolism (e.g. *g6pc3*, *tigara*).

### Validation of temperature-induced growth changes in mRNA expression levels of candidate genes by qPCR

**Fig. 3.**
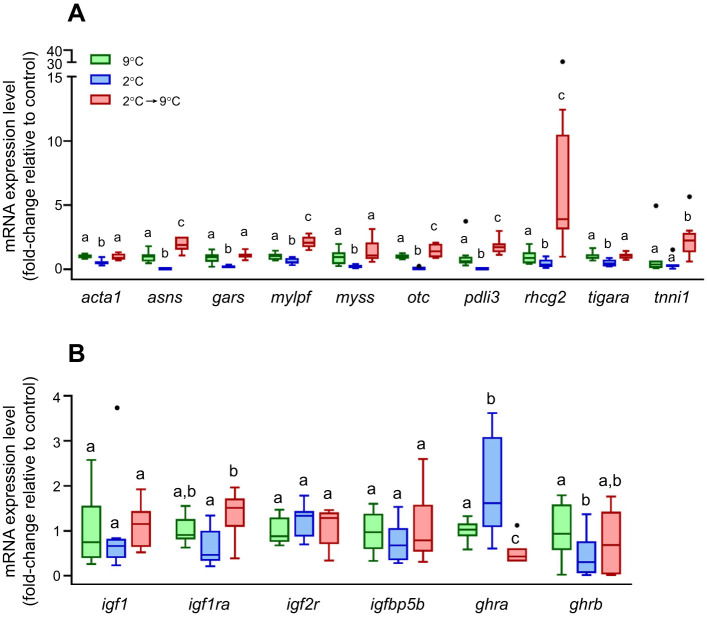
**Effects of temperature-induced growth manipulations on mRNA expression levels in white muscle.** (A) DEGs identified by RNAseq. (B) Candidate genes involved in insulin-like growth factor 1 (IGF1)- and growth hormone (GH)-regulated muscle growth. Results are expressed as fold-change relative to the control group (9°C), which was set to 1. Box plots are described in the legend to Fig. 1 (*N*=8). Statistically significant (*P*<0.05, Dunn's *post hoc*) differences among groups are indicated by different letters.

We validated the observed transcriptomic changes by measuring the mRNA expression levels of a subset of ten common DEGs by qPCR. For all genes examined, mRNA expression levels were lower (*P*<0.05) in the 2°C group (growth suppression) than in the 9°C group, with the exception of troponin I (*tnni1*) (Fig. 3A). Furthermore, the mRNA expression levels of most DEGs examined were higher in the 2°C→9°C group (growth stimulation) than in the 9°C group, although only significantly (*P*<0.05) for *asns*, *mylpf*, *otc*, *pdli3*, *rhcg2* and *tnni1* (Fig. 3A). We also measured the expression levels of known genes involved in insulin-like growth factor 1 (IGF1)- and growth hormone (GH)-regulated muscle growth (Mommsen and Moon, 2001; Wood et al., 2005): *igf1* and its receptor a (*igf1ra*), IGF2 receptor (*igf2r*), IGF binding protein 5b (*igfbp5b*), and GH receptors a and b (*ghra* and *grhb*, respectively) (Fig. 3B). The mRNA expression levels of *igf1ra*, *igfbp5b* and *grhb* were lower in the 2°C treatment group than in the 9°C group, although only significantly (*P*<0.05) for *ghrb*. Furthermore, *igf1ra* was the only gene that showed higher (*P*<0.05) mRNA expression levels in the 2°C→9°C group than in the 9°C group. In contrast, the mRNA expression levels of *grha* were higher (*P*<0.05) in the 2°C group than in the 9°C group, and lower (*P*<0.05) in the 2°C→9°C group than in the 9°C or 2°C groups (Fig. 3B).

### White muscle proteomic profiling of temperature-induced growth manipulations

**Fig. 4.**
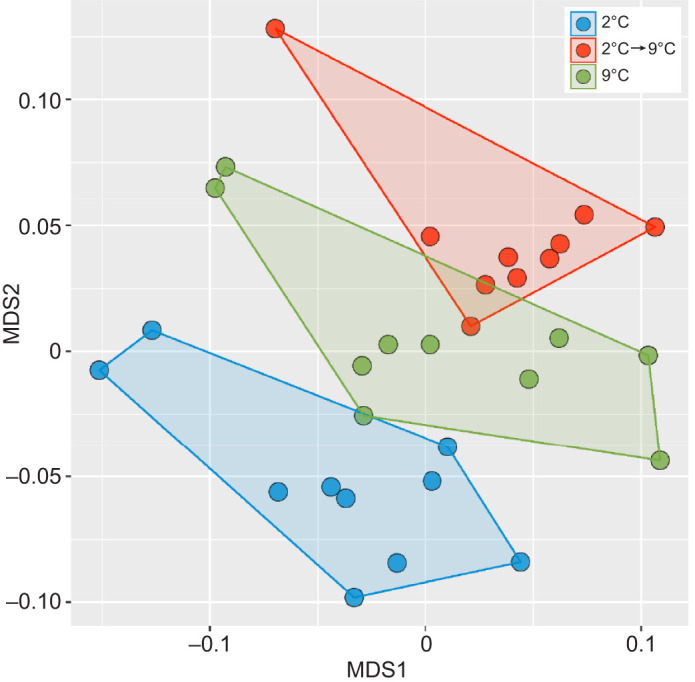
**Non-metric multidimensional scaling plot (NMDS) of white muscle proteomic profiles.** White muscle proteomes differ among juvenile fish by temperature regime (*P*<0.05).

Label-free shotgun proteomics was performed to identify DAPs in white muscle of juvenile Pacific halibut that respond to temperature-induced growth manipulations. Proteome-level data of individual white muscle samples clustered by the experimental temperature regime, with samples from the 9°C group (control) separated from those of the 2°C (growth suppression) and the 2°C→9°C (growth stimulation) groups (Fig. 4). ANOSIM analyses revealed significant differences in protein abundance among the three experimental groups (*R*=0.4253; *P*=0.001). Furthermore, ANOSIM pairwise comparisons evidenced significant differences in protein abundance in the two temperature-induced growth manipulations: growth suppression (9°C versus 2°C; *R*=0.2593; *P*=0.001) and growth stimulation (9°C versus 2°C→9°C; *R*=0.2224; *P*=0.005).

Quantitative analyses of proteomic data from fish under growth suppression, as conducted through pairwise comparison of the 9°C and the 2°C groups, identified 241 DAPs, with 91 and 150 proteins at increased and decreased abundance, respectively, in the 2°C group relative to the 9°C group (Fig. 2B; Tables S2 and S7). In addition, analyses of proteomic data from fish under growth stimulation identified 159 DAPs, with 149 and 10 proteins at increased and decreased abundance, respectively, in the 2°C→9°C group relative to the 9°C group (Fig. 2B; Tables S2 and S8). GO enrichment analysis of proteins with decreased abundance under growth suppression revealed a significant over-representation of biological process terms related primarily to translation, peptide and macromolecule biosynthetic processes, protein and nucleic acid metabolic processes, and gene expression (Table S9). Top DAPs with decreased abundance under growth suppression include proteins involved in protein translation (e.g. Eef1e1, Farsa, Iars1, If2b, Kars1, Mars1, Nars1, Rars1, Yars1) and amino acid metabolism (Asns) (Table S7). (Full protein names are given in Table 2 and Tables S7 and S8. Protein nomenclature according to https://zfin.org.) Also with decreased abundance are actin-binding proteins that participate in muscle sarcomeric function and development (e.g. Myo18A, Myoz2a, Smtnl2, Tgfbi, Tnni1), and transcriptional regulation (e.g. Klhl31, Setd3). In comparison, despite a smaller number of biological process GO terms identified, enrichment analysis of proteins with increased abundance under growth stimulation produced GO terms related to amino acid biosynthetic process and, although not significantly, protein transport and localization (Table S10). Among the top DAPs with increased abundance under growth stimulation are proteins involved in protein translation, synthesis and transport (e.g. Abcf2a, Copb1, Drg2, Eef2k, Ipo4, Psat1, Tsr1, Xpo1b) (Table S8). Other up-regulated DAPs include proteins involved in muscle development (e.g. Obsl1, Pdlim3a, Xirp1), transcriptional regulation (Apex1), regulation of cellular growth (Csnk2a1), cell adhesion (Fn1b) and apoptosis regulation (Api5).

**Table 2.** List of common differentially abundant proteins (DAPs) in white skeletal muscle with decreased abundance under growth suppression (9°C vs 2°C) and increased abundance under growth stimulation (9°C vs 2°C→9°C)

UniProt ID	UniProt name	UniProt description	Protein name
A0A0F8AMT8	ASNS_CHICK	Asparagine synthetase [glutamine-hydrolyzing]	Asns
A0A0F8CHX9	W5U780_ICTPU	ATP-binding cassette sub-family F member 2	Abcf2
A0A0F8D1Y1	COPG2_DANRE	Coatomer subunit gamma	Copg2
I3KF30	I3KF30_ORENI	Cysteinyl-tRNA synthetase	Cars1
I3JQ92	I3JQ92_ORENI	D-3-phosphoglycerate dehydrogenase (EC1.1.1.95)	Phgdh
I3JBA9	I3JBA9_ORENI	Dynein cytoplasmic 1 heavy chain 1	Dync1h1
B5X3A9	B5X3A9_SALSA	Eukaryotic translation initiation factor 2 subunit 2	Eif2s2
H3CVI5	H3CVI5_TETNG	Eukaryotic translation initiation factor 3 subunit J	Eif3J
G3Q1D7	G3Q1D7_GASAC	Eukaryotic translation termination factor 1a	Etf1
I3J5Q8	I3J5Q8_ORENI	Fibronectin 1b	Fn1
G3Q6K3	G3Q6K3_GASAC	Heat shock protein 14	Hspa14
I3KBQ3	I3KBQ3_ORENI	Immunoglobulin-like and fibronectin type III domain containing 1	Igfn1
G3PSV2	G3PSV2_GASAC	Importin 7	Ipo7
E6ZID3	E6ZID3_DICLA	Importin-4	Ipo4
A0A146VMN7	LAR1B_HUMAN	La-related protein 1B	Larp1b
A0A1A8I6E2	MAP2_HUMAN	Methionine aminopeptidase (EC3.4.11.18)	Metap2
G3NT96	SYMC_HUMAN	Methionyl-tRNA synthetase	Mars1
I3K086	I3K086_ORENI	N(alpha)-acetyltransferase 15	Naa15
A0A1A8PGE0	NAA50_DANRE	N(Alpha)-acetyltransferase 50	Naa50
M4ABS1	M4ABS1_XIPMA	Ornithine carbamoyltransferase	Otc
I3KJY9	I3KJY9_ORENI	Phosphoserine aminotransferase (EC2.6.1.52)	Psat1
Q45TX4	Q45TX4_PAROL	Pigment epithelium-derived factor	Serpinf1
M3ZE42	M3ZE42_XIPMA	Protein arginine N-methyltransferase 5 (EC2.1.1)	Prmt5
E6ZHM9	E6ZHM9_DICLA	Protein BCCIP homolog	Bccip
A0A0F8B866	G3BP1_MOUSE	Ras GTPase-activating protein-binding protein 1	G3bp1
H2TH42	H2TH42_TAKRU	Reticulocalbin 3	Rcn3
I3K6L3	I3K6L3_ORENI	S-adenosylmethionine synthase (EC2.5.1.6)	Mat2a
A0A2I4AXM8	Q7SXN1_DANRE	Serine hydroxymethyltransferase (EC2.1.2.1)	Shmt1
A0A0F8BRK7	SMTL2_HUMAN	Smoothelin-like protein 2	Smtnl2
A0A0F8BWW2	I3J2J3_ORENI	Transmembrane protein 161A	Tmem161a
I3IZT0	I3IZT0_ORENI	Ubiquitin carboxy-terminal hydrolase	Usp13
G3PJV2	G3PJV2_GASAC	Eukaryotic translation initiation factor 4 gamma 1a	Eif4g1

Protein nomenclature according to https://zfin.org.

We also set out to identify DAPs that changed in the same direction as the observed changes in SGR in the two types of temperature-induced manipulations by comparing DAPs with decreased abundance under growth suppression with DAPs with increased abundance under growth stimulation. We identified 32 common DAPs (Fig. 2B, Table 2) involved primarily in amino acid and protein synthesis and translation (e.g. Abcf2, Asns, Cars1, Eif2s3, Eif3J, Eif4g1, Etf1, Hspa14, Mars1, Mat2a, Metap2, Otc, Phgdh, Psat1, Rcn3, Shmt1), and protein transport and metabolism (e.g. Copg2, Naa15, Usp13).

### Parallel transcriptomic and proteomic responses in white muscle to temperature-induced growth manipulations

To identify parallel responses at the transcriptomic and proteomic levels, we next set out to identify DEGs that also showed significant changes in abundance at the protein level in the same direction as the observed changes in SGR for each of the temperature-induced growth manipulations. First, growth suppression resulted in a significant decrease at both the mRNA expression and protein abundance levels of 24 genes (Table 3) known to be involved in amino acid and protein metabolism, including protein synthesis and translation (*asns*, *eef1d*, *eif2s2*, *eif3ja*, *eif4g2*, *farsa*, *fkbp7*, *gars*, *oct*, *psat1*, *rars*), protein transport (*ipo13*), muscle function, development and contraction (*cap2*, *hspb11*, *mpst*, *myoz2*, *setd7*, *tnni2*), actin filament function and stabilization (*coro1a*, *ivns1abpa*), collagen formation (*col1a2*, *col5a1*, *col6a6*), and ribosome biogenesis (*bccip*). Second, growth stimulation resulted in a significant increase at both the mRNA expression and protein abundance levels of 6 genes (Table 3) known to be involved in amino acid and protein synthesis, translation and metabolism (*asns*, *metap2*, *oct*), muscle contraction and development (*igfn1*, *ttn*), and collagen stabilization (*phy2*). Importantly, we identified two genes that changed their expression at both the mRNA and protein levels in the same direction as the change in SGR: *asns* and *otc*.

**Table 3.** List of matched DEGs and DAPs in white muscle that are down-regulated in response to growth suppression (9°C vs 2°C) and up-regulated in response to growth stimulation (9°C vs 2°C→9°C)

UniProt ID	UniProt name	UniProt description	Gene name
**Down-regulated under growth suppression**			
P25325	THTM_HUMAN	3-mercaptopyruvate sulfurtransferase	*mpst*
Q5R5X8	CAP2_PONAB	Adenylyl cyclase-associated protein 2	*cap2*
P40329	SYRC_RAT	Arginine--tRNA ligase, cytoplasmic	*rars*
P49088	ASNS_RAT	Asparagine synthetase [glutamine-hydrolyzing]	*asns*
O88207	CO5A1_MOUSE	Collagen alpha-1(V) chain	*col5a1*
P02467	CO1A2_CHICK	Collagen alpha-2(I) chain	*col1a2*
A6NMZ7	CO6A6_HUMAN	Collagen alpha-6(VI) chain	*col6a6*
P31146	COR1A_HUMAN	Coronin-1A	*coro1a*
P29693	EF1D_XENLA	Elongation factor 1-delta	*eef1d*
P20042	IF2B_HUMAN	Eukaryotic translation initiation factor 2 subunit 2	*eif2s2*
Q7SXU0	EI3JA_DANRE	Eukaryotic translation initiation factor 3 subunit J-A	*eif3ja*
Q62448	IF4G2_MOUSE	Eukaryotic translation initiation factor 4 gamma 2	*eif4g2*
P41250	SYG_HUMAN	Glycine-tRNA ligase	*gars*
A5JV83	HSPBB_DANRE	Heat shock protein beta-11	*hspb11*
Q6DHG0	SETD7_DANRE	Histone-lysine N-methyltransferase	*setd7*
Q5R974	IPO13_PONAB	Importin-13	*ipo13*
Q5RG82	NS1BA_DANRE	Influenza virus NS1A-binding protein homolog A	*ivns1abpa*
Q5R6I2	MYOZ2_PONAB	Myozenin-2	*myoz2*
P00480	OTC_HUMAN	Ornithine carbamoyltransferase, mitochondrial	*otc*
O54998	FKBP7_MOUSE	Peptidyl-prolyl cis-trans isomerase FKBP7	*fkbp7*
Q1JPX3	SYFA_DANRE	Phenylalanine--tRNA ligase alpha subunit	*farsa*
Q99K85	SERC_MOUSE	Phosphoserine aminotransferase	*psat1*
Q5BJJ7	BCCIP_DANRE	Protein BCCIP homolog	*bccip*
P02643	TNNI2_RABIT	Troponin I, fast skeletal muscle	*tnni2*
**Up-regulated under growth stimulation**			
Q5ZJU3	ASNS_CHICK	Asparagine synthetase [glutamine-hydrolyzing]	*asns*
Q86VF2	IGFN1_HUMAN	Immunoglobulin-like and fibronectin type III domain-containing protein 1	*igfn1*
P50579	MAP2_HUMAN	Methionine aminopeptidase 2	*metap2*
P00480	OTC_HUMAN	Ornithine carbamoyltransferase, mitochondrial	*otc*
Q20065	P4HA2_CAEEL	Prolyl 4-hydroxylase subunit alpha-2	*phy2*
Q8WZ42	TITIN_HUMAN	Titin	*ttn*

Gene nomenclature according to https://zfin.org.

### White muscle stable isotope analyses

**Fig. 5.**
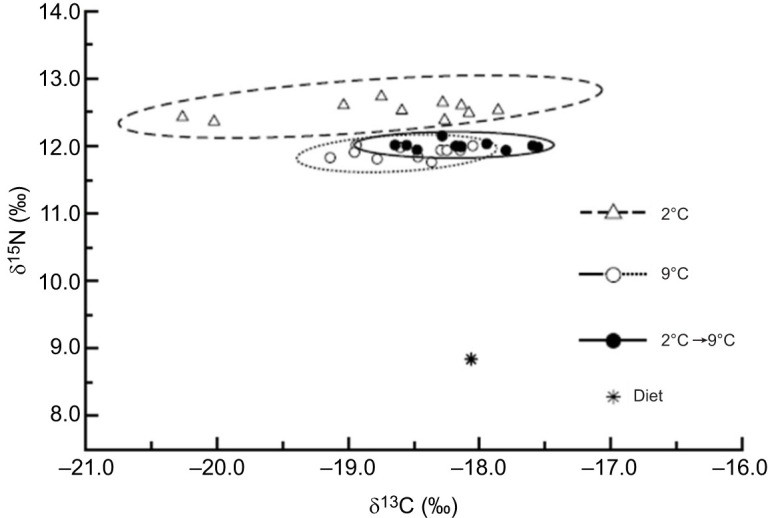
**Bayesian standard ellipses for each of the temperature-induced growth modification groups.** Δ^15^N values (means±s.d.) were 3.69±0.12, 3.07±0.08 and 3.17±0.06 for the 2°C, 9°C and 2°C→9°C groups, respectively (*F*_1,30_=24.71, *P*<0.05). Δ^13^C values (means±s.d.) were −0.66±0.82, −0.48±0.36 and −0.05±0.37 for the 2°C, 9°C and 2°C→9°C groups, respectively (*F*_1,30_=0.03, *P*=0.74).

To further understand growth-associated physiological changes in skeletal muscle metabolism, we conducted ^15^N and ^13^C stable isotope analyses of white muscle from fish subjected to temperature-induced growth manipulations. Bayesian standard ellipses for the three groups differed in area, overlap and position (Fig. 5). The total area of the 2°C group ellipse was larger than those of the 9°C and the 2°C→9°C groups (Table S11). This difference was driven primarily by a comparatively large range in ^13^C values within the 2°C group. Furthermore, the 2°C group ellipse did not overlap with those of either of the other groups, although the 9°C and the 2°C→9°C group ellipses demonstrated modest overlap (Table S12). Mean Δ^15^N values were significantly different (*P*<0.05) among the three groups (Fig. 5), with larger values observed for the 2°C group and substantially similar values for the 9°C and the 2°C→9°C groups, as supported by analysis of polar vectors among ellipse centroids (Table S12). Mean Δ^13^C values were not significantly different (*P*=0.74) among groups, mainly because of large variability within the 2°C treatment group (Fig. 5). Mean C:N ratios were not significantly different among groups (*P*=0.29), but the 2°C group demonstrated more variability than either of the other two treatment groups (Table S13).

### Candidate gene expression patterns in relation to fish size in wild Pacific halibut

**Fig. 6.**
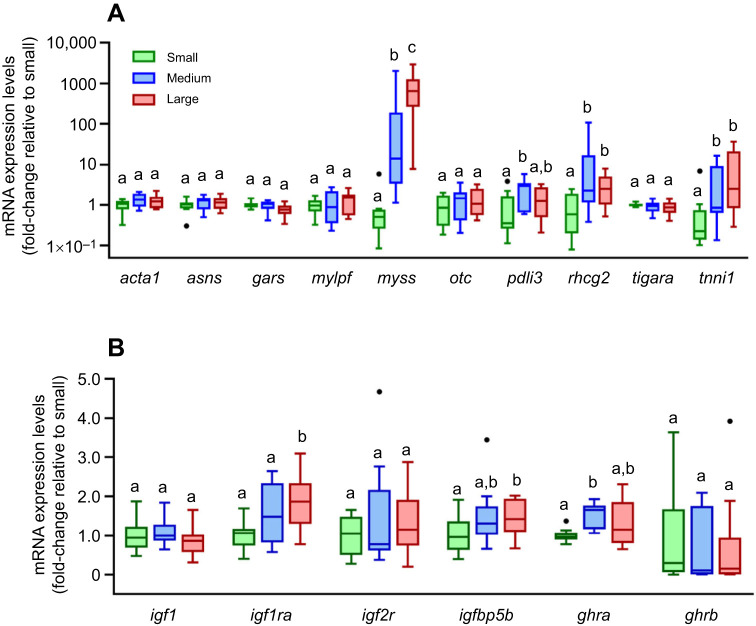
**Comparison of mRNA expression levels in white muscle of age-matched juvenile Pacific halibut of three different size categories (small, medium, large).** (A) DEGs identified by RNAseq. (B) Candidate genes involved in IGF1- and GH-regulated muscle growth. Results are expressed as fold-change relative to the small group, which was set to 1. Box plots are described in the legend to Fig. 1 (*N*=10). Statistically significant (*P*<0.05, Dunn's *post hoc*) differences among groups are indicated by different letters.

Given the identification and validation of a set of common DEGs that responded to temperature-induced growth manipulations, we investigated whether their expression levels could be used to infer possible growth differences in relation to fish size-at-age. To approach this, we measured the mRNA expression levels of these genes by qPCR in white muscle from wild-captured, age-matched (4-year-old) juvenile Pacific halibut of three different size categories (small, medium and large) (Fig. S1A). No differences in length were observed between males and females in either of the three size groups (Fig. S1B), discarding possible sex-related growth differences. We observed higher (*P*<0.05) mRNA expression levels for *myss*, *pdli3*, *rhcg2* and *tnni1* in fish in the medium and/or large size groups than in the small size group (Fig. 6A). Higher (*P*<0.05) mRNA expression levels for *igf1ra*, *igfbp5a* and *ghra* were also detected in medium and/or large size groups over the small size group (Fig. 6B).

## DISCUSSION

While growth plasticity in fish is well known to occur in response to thermal variation (Crozier and Hutchings, 2014; Jobling, 1996), the mechanisms involved are not completely understood. The present study describes for the first time molecular processes at the gene, protein and metabolic levels that underly thermally induced growth plasticity in juvenile Pacific halibut and identifies a set of muscle growth biomarkers for thermally induced growth changes that can capture somatic growth variation in wild Pacific halibut. These growth biomarkers will be useful to elucidate the influence of factors driving somatic growth variation, including changes in size-at-age, in Pacific halibut.

Our results indicate that low temperature acclimation results in a suppression of growth, whereas acclimation to a favorable thermal environment following a period of growth suppression results in growth stimulation known as compensatory growth (Ali et al., 2003). The observed ability of temperature to modulate growth rates in juvenile Pacific halibut confirms the results of previous studies (Hurst et al., 2005) and reinforces the notion of the plastic nature of growth in juveniles of this species.

Our transcriptomic and proteomic data indicate that changes in somatic growth are strongly associated with changes in the protein synthesis machinery in white muscle. This is evidenced by the observed parallel changes in the mRNA expression and abundance of genes and proteins, respectively, with biological functions related to amino acid and protein synthesis, translation, protein transport and protein metabolism. In our study, several protein synthesis processes appear to be down-regulated during temperature-induced growth suppression and up-regulated during temperature-induced compensatory growth. These common processes include factors that participate in key stages in protein synthesis such as translation initiation (e.g. *eif2s2*, *eif3ja*, *eif4g2*, *farsa*, *gars*, *rars*), elongation (e.g. *eef1d*, *rpl22*) and termination (e.g. *abce1*), as well as factors involved in amino acid biosynthesis (e.g. *asns*, *psat1*, *metap2*), and in the prevention of protein misfolding (*fkbp7*) and ubiquitin-mediated protein degradation (*usp13*). A number of these same processes and factors involved in protein synthesis are also associated with fast growth or with growth performance in a wide variety of fish species, including flatfish, as evidenced by studies using transcriptomic and proteomic approaches similar to those used in the present study (Causey et al., 2019; Gabriel Kuniyoshi et al., 2019; Gayo et al., 2024; Li et al., 2022; Palstra et al., 2013, 2014; Rescan et al., 2017; Yang et al., 2019).

Further evidence in support of the importance of white muscle protein synthesis underlying changes in growth rate is derived from the incorporation of naturally occurring stable carbon and nitrogen isotopes into white muscle. Previous studies have shown that growth can play a disproportionately large role in influencing the speed and manner in which dietary carbon and nitrogen isotopes are incorporated into the skeletal muscle tissue of ectotherms compared with metabolic replacement (Nahon et al., 2020; Perga and Gerdeaux, 2005; Reich et al., 2008). Consequent to this dominance, temperatures that promote increased somatic growth rates also promote faster incorporation of both carbon and nitrogen isotopes (Bloomfield et al., 2011). Our results show lower Δ^15^N in the control (9°C) group than in the growth suppression group, consistent with efficient routing of dietary amino acids to facilitate protein anabolism associated with rapid somatic growth (Barreto-Curiel et al., 2018; Busst and Britton, 2018). Similar inverse relationships between growth rate and δ^15^N in white muscle have been reported for gilthead sea bream (*Sparus aurata*) (Martin-Perez et al., 2013) and Atlantic salmon (*Salmo salar*) (Trueman et al., 2005).

It is worth noting that the pattern of elevated δ^15^N in accumulated tissues during temperature-induced compensatory growth was sufficient to offset differences in Δ^15^N resulting from the prior low temperature acclimation. Mean Δ^13^C, in contrast, did not differ significantly in response to temperature-induced growth manipulations, mainly resulting from large variability among fish experiencing growth suppression. Previous studies have observed effects of temperature on Δ^13^C (Barnes et al., 2007; Britton and Busst, 2018), ostensibly resulting from the effects of both temperature (Farkas et al., 2001) and growth (Barreto-Curiel et al., 2018) on tissue lipid concentrations. Indeed, using C:N ratio as a rough proxy for tissue lipid content (Barnes et al., 2007), we observed substantially greater variability among fish experiencing growth suppression, paralleling the variability in Δ^13^C, whereas fish experiencing compensatory growth had the lowest C:N ratio, presumably resulting from the catabolism of lipids in white muscle to support the increase in growth. The observed decrease in HSI in fish experiencing compensatory growth points to the mobilization of liver lipids for their catabolism in skeletal muscle, as these are the first energetic reserves that are metabolized in fish (Black and Love, 1986), and to the important role of liver-derived energy for skeletal muscle growth (Mommsen and Moon, 2001). As observed for juvenile Pacific halibut in this and previous studies (Hurst, 2004), an inverse relationship between acclimation temperature and HSI has also been reported for other fish species (Blier and Guderley, 1988; Kent et al., 1988). Although we did not identify clear transcriptomic or proteomic signals of lipid catabolism in skeletal muscle of fish undergoing compensatory growth, we cannot rule out that changes in lipid catabolism may have taken place at the enzyme activity level. Overall, these results indicate that temperature-induced growth manipulations resulted in changes in white muscle isotope composition, as evidenced primarily by changes in δ^15^N and, consequently, protein metabolism, as almost all N is derived from the protein fraction (Wood, 2001). These results provide direct functional support to the identified changes in constituents of the translation and protein synthesis machinery at the transcriptomic and proteomic levels and strongly suggest that protein synthesis in white muscle is the major process underlying growth changes in Pacific halibut. This is consistent with the well-established notion that growth in fish occurs under conditions that lead to a net gain in protein synthesis in white muscle (Houlihan et al., 1995).

Importantly, our results show that growth-associated changes in protein synthesis in white muscle are accompanied by changes in the mRNA expression and abundance of genes and proteins, respectively, that constitute structural and contractile elements present in myofibers. These include factors present in the sarcomere (i.e. the functional unit of myofibers) such as *acta*, *myss*, *mylpf*, *myoz2*, *tnni1* and *ttn*, and factors involved in muscle development (e.g. *igfn1*, *lrrc2*, 3*mpst*, *cap2*, *hspb11*, *setd7*). These results support the notion that the synthesis of myofibrillar protein represents the largest contributor to protein synthesis in the growing fish muscle (Carter and Houlihan, 2001; Mommsen, 2001), and strongly point to this process as the primary mechanism driving the hypertrophic growth of muscle fibers that characterizes post-embryonic somatic growth in fish (Johnston, 2006; Johnston et al., 2011). Again, a number of these muscle structural and regulatory factors have also been previously reported to vary their mRNA expression and protein abundance levels in other fish species, including flatfish, in relation to growth patterns in several transcriptomic and proteomic studies that also reported parallel molecular changes in the protein synthetic machinery (Causey et al., 2019; Li et al., 2022; Mendez et al., 2018; Palstra et al., 2013, 2014; Rescan et al., 2017; Xu et al., 2017; Yang et al., 2019). Therefore, our results indicate that somatic growth in juvenile Pacific halibut is primarily due to the increase of myofibrillar protein synthesis in white muscle. Somatic growth is also associated with the increase in mRNA expression and protein abundance of factors involved in collagen synthesis (e.g. *col1a2*, *col5a1*, *col6a6*, *phy2*), supporting the results of previous studies on other fish species under growth-stimulating conditions (Cao et al., 2022; Dong et al., 2022; Palstra et al., 2013, 2014). Collagen is a key factor in the development of the connective tissue that surrounds the muscle fibers (Bremner and Hallett, 1985) and, therefore, our results suggest that white muscle growth requires the concurrent synthesis of collagen and myofibrillar protein. This is likely important to ensure proper assembly and contractile functionality of muscle fibers within the growing skeletal muscle in juvenile Pacific halibut.

A particularly relevant outcome of our transcriptomic analysis is the identification of individual genes that change their mRNA expression levels in white muscle in the same direction of growth changes as these genes represent potential molecular markers for growth that can be measured with the use of established molecular techniques (i.e. qPCR). Among potential growth marker genes further validated by qPCR are genes involved in protein translation initiation (*gars*) and amino acid anabolism (*asns*), previously shown to be upregulated in muscle by dietary manipulation in juvenile turbot (*Scophthalmus maximus*) (Xu et al., 2017) and to be associated with protein deposition in carp (*Cyprinus carpio*) skeletal muscle (Wu et al., 2024). Interestingly, two potential growth marker genes are involved in nitrogen excretion: *otc*, encoding a key enzyme in the ornithine–urea cycle that helps detoxify ammonia, produced as a result of amino acid catabolism, via ureagenesis (Todgham et al., 2001), and *rhcg2*, encoding a transporter that allows ammonia to leave muscle cells, preventing its accumulation in skeletal muscle. Free amino acids are important substrates for the synthesis of essential biomolecules for fish larval and juvenile growth (Wright and Fyhn, 2001). The observed increase in mRNA expression levels of *otc* and *rhcg2* in white muscle of juvenile Pacific halibut experiencing compensatory growth is consistent with the notion that temperature-driven increases in protein synthesis occur in parallel to nitrogen excretion primarily in the form of ammonia (Wood, 2001). It is worth noting that *asns* and *otc* changed their expression in the direction of the growth change at both the mRNA and protein levels. Other potential growth marker genes encode structural and regulatory elements in the muscle sarcomere that play a key role in muscle contraction and growth: *acta1*, the main actin component of the thin filaments, and *myss* and *mylpf*, two myosin isoforms that make up the thick filaments (Clark et al., 2002; Joyce, 2023). Furthermore, *tnni1* and *pdli3* encode sarcomeric proteins that modulate the interaction of myosin and actin during contraction and stabilize the muscle cytoskeleton during muscle fiber contraction and growth, respectively (Clark et al., 2002; Kluever et al., 2011). Another potential growth marker gene is *tigara*, encoding a bifunctional enzyme involved in glucose metabolism that is important for skeletal muscle differentiation and a marker for mature skeletal muscle (Vandoolaeghe et al., 1999).

In addition to these potential growth marker genes identified by transcriptomic profiling, we found potential growth marker genes among known candidate genes that belong to the GH/IGF1 pathway, a central regulator of growth and protein synthesis in skeletal muscle of all vertebrates (Mommsen and Moon, 2001; Wood et al., 2005). *igf1ra* encodes the specific receptor for Igf1, a growth factor largely produced in the liver in response to Gh stimulation that acts directly on skeletal muscle to stimulate protein synthesis and hypertrophic growth in fish (Mommsen and Moon, 2001). Our results suggest that changes in *igf1ra* mRNA expression levels in response to temperature-induced growth variation may alter the responsiveness of juvenile Pacific halibut white muscle to the growth-promoting actions of Igf1. In support of a role for *igf1ra* in skeletal muscle growth, *igf1ra* mRNA expression levels in white muscle are correlated with nutritional state and/or growth rate in juvenile flatfish (Hagen et al., 2009; Østervold et al., 2025) and in non-flatfish species (Chauvigné et al., 2003; Montserrat et al., 2007). Furthermore, the two paralogous genes *ghra* and *ghrb* (Ocampo Daza and Larhammar, 2018) changed their mRNA expression levels in response to temperature-induced growth manipulations in a completely opposite manner. On the one hand, *ghra* showed an inverse relationship between mRNA expression levels and growth similar to that observed in Atlantic salmon (*Salmo salar*) post-smolt (Hevrøy et al., 2013); on the other hand, *ghrb* changed its mRNA expression levels in the direction of temperature-induced growth variation. Regardless of their potential value as growth markers, the different expression patterns of these two paralogous genes suggest that they may differ functionally. Based on previous studies relating *ghr* mRNA expression levels in salmonid white muscle to the known lipolytic activity of Gh (Hevrøy et al., 2013, 2015), we speculate that Gh-mediated lipid catabolism in the growing muscle may be facilitated by *ghrb* expression. Clearly, further studies are needed to better understand the potential role of these two paralogous Gh receptor genes in regulating skeletal muscle growth.

Notably, a subset of these potential growth marker genes (*myss*, *pdli3*, *rhcg2*, *tnni1*, *igf1ra*, *igfbp5a* and *ghra*) also differed in their mRNA expression levels in white muscle in relation to the size of age-matched Pacific halibut juveniles collected in the wild, irrespective of sex. All these seven genes showed significantly higher mRNA expression levels in medium and/or large individuals than in small individuals, suggesting that size-dependent expression of these potential growth marker genes may indicate that size differences among age-matched individuals are likely the result of differences in their growth rate. Therefore, these growth marker genes could assist in determining whether phenotypic variation in size can be explained by somatic growth differences among individuals, as reflected by differences in growth marker gene expression. Furthermore, this set of growth marker genes may prove useful in future studies that investigate the effects of growth-influencing factors driving the well-known temporal and spatial variation in size-at-age in Pacific halibut (Clark et al., 1999; Ritchie et al., 2025).

In summary, this study provides insights into the molecular mechanisms that underlie growth plasticity in juvenile Pacific halibut in response to thermal variation. Our results show that growth plasticity involves the activation of tiered plastic molecular responses at the gene, protein and metabolic levels in skeletal muscle, with all these responses finely tuned to regulate the synthesis of myofibrillar proteins, among other muscle-related processes. Upregulation of myofiber-related encoding genes and proteins under thermal conditions promoting growth may increase individual fitness through increased muscle mass and, consequently, body size. These processes are likely common to other fish species exhibiting plastic growth. Furthermore, we have identified and validated a set of individual growth biomarker genes from the laboratory experiment that, when tested under field conditions, show differential expression relative to fish size in wild individuals. These growth biomarkers, that include known and novel growth-related genes, could be potentially used to infer the type of growth pattern from size and to elucidate the influence of factors driving somatic growth variation, including changes in size-at-age. Overall, the results of the present study improve our mechanistic understanding of growth plastic responses to variable temperature regimes in teleost fish and highlight their potential for resilience and/or adaptability in the face of environmental variability. For Pacific halibut, this is supported by recent modeling studies projecting relatively moderate changes in biomass throughout the northeast Pacific Ocean in response to marine heatwaves and future climate scenarios (Cheung and Frölicher, 2020; English et al., 2022; Thompson et al., 2023).

## Supplementary Material

10.1242/jexbio.251013_sup1 Supplementary information

Table S3. List of down-regulated differentially expressed genes (DEGs) in white skeletal muscle in response to growth suppression by low-temperature acclimation (9^°^C vs 2^°^C).

Table S4. Enrichment of Gene Ontology classes of down-regulated differentially expressed genes (DEGs) in white skeletal muscle identified in response to growth suppression by low-temperature acclimation (9^°^C vs 2^°^C).

Table S5. List of up-regulated differentially expressed genes (DEGs) in white skeletal muscle in response to growth stimulation (9^°^C vs 2^°^C→9^°^C).

Table S6. Enrichment of Gene Ontology classes of up-regulated differentially expressed genes (DEGs) in white skeletal muscle identified in response to growth stimulation (9^°^C vs 2^°^C→9^°^C).

Table S7. List of differentially abundant proteins (DAPs) in white skeletal muscle in response to growth suppression by low-temperature acclimation (9^°^C vs 2^°^C).

Table S8. List of differentially abundant proteins (DAPs) in white skeletal muscle in response to growth stimulation (9^°^C vs 2^°^C→9^°^C).

Table S9. Enrichment of Gene Ontology classes of differentially abundant proteins (DAPs) in white skeletal muscle identified in response to growth suppression by low-temperature acclimation (9^°^C vs 2^°^C).

Table S10. Enrichment of Gene Ontology classes of differentially abundant proteins (DAPs) in white skeletal muscle identified in response to growth stimulation (9^°^C vs 2^°^C→9^°^C).
