# Supplementary material for: Mechanisms underlying thermally induced growth plasticity in juvenile Pacific halibut
Source: J Exp Biol. 2025 Oct 6;228(19):jeb251013. doi: 10.1242/jeb.251013 (PMC12539203; doi:10.1242/jeb.251013)
Supplement: Supplementary information [file jexbio-228-251013-s1.pdf]

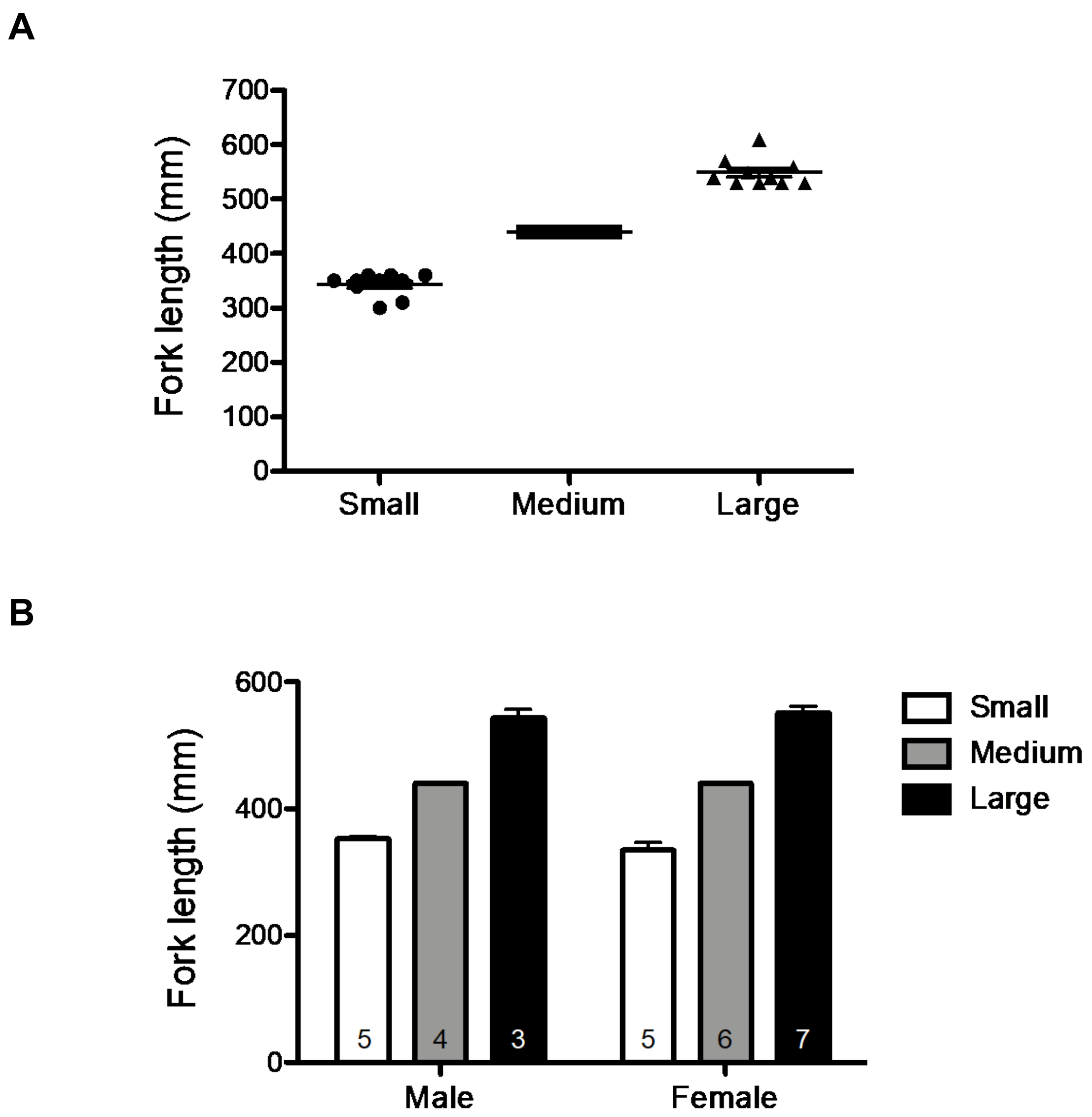


**Fig. S1.** Fork length (mm) of age-matched juvenile Pacific halibut in the small, medium and large size categories (average is shown by a horizontal line) (A). Fork length (mm) of male and female juvenile Pacific halibut by size category. Bars represent the mean ± SEM and numbers indicate the sample size (B).

**Table S1.** Sequences of primers used in gene expression analyses by real-time quantitative PCR.

| Accession No. | Gene name | Primer sequence (5' to 3') | Amplicon size (bp) |
|---|---|---|---|
| XM_020084913.1 | Alpha actin (*acta1*) | (F) ACGACGAAGAAACTACCGCC<br>(R) CCATACCGACCATGACACCC | 141 |
| XM_020113072.1 | Ammonium transporter Rh type C2 (*rhcg2*) | (F) CCATCCCGCCTGTTTTGG<br>(R) CGATCTCCTGTTGTGCCG | 109 |
| XM_020090379.1 | Asparagine synthetase (*asns*) | (F) GTTTCCTTTCTTGTGGCTCTGC<br>(R) CAAAGCGGTCATACAGGTGG | 128 |
| XM_020079459.1 | Probable fructose 2,6-bisphosphatase (*tigara*) | (F) GGAGTTGCTCTGTTCTTTGCC<br>(R) GTCCTCCTCTTTCCTCACGC | 109 |
| EB032115.1 | Glyceraldehyde-3-phosphate dehydrogenase (*gapdh*) | (F) CGACCTGATGCTCCACATGG<br>(R) GGAGAACGTGGGTGATGTGG | 87 |
| XM_035142542.1 | Glycine-tRNA ligase (*gars*) | (F) GACCTTCAAACTCACCAAGGA<br>(R) TGCCAAAGGAGGGTTCAATTA | 101 |
| XM_035165049.1 | Growth hormone receptor a (*ghra*) | (F) GCCTCATTTCACTGACTGCG<br>(R) AGGTAGAAGACTCGGAGTGC | 111 |
| XM_035184115.1 | Growth hormone receptor b (*grhb*) | (F) CGACGAGGACCTGTTCTACG<br>(R) GGAACTCACATTCAGCAGCG | 85 |
| XM_035147552.1 | Insulin-like growth factor 1(*igf1*) | (F) TGGACGAGTGCTGCTTCC<br>(R) TCTTGTCTGGCTGCTGTGC | 195 |
| KF917549.1 | Insulin-like growth factor binding protein 5b (*igfbp5b*) | (F) GAGCAGAAGAGGAAGCAGGG<br>(R) AAGGACAGAGCCATGACACG | 164 |
| XM_035156283.2 | Insulin-like growth factor 1 receptor a (*igf1ra*) | (F) TGCTGCTGAGATGTACGTCC<br>(R) ATGGTGATCTTCTCCCTCGC | 57 |
| XM_035143619.1 | Insulin-like growth factor 2 receptor (*igf2r*) | (F) GCTCCCAGTACAACAACAAGG<br>(R) GCGTAGGATGTGTACCAGTGG | 142 |
| XM_034593748.1 | Myosin Heavy Chain (myss) | (F) TTGTTCTTCTCCAGCCAGCC<br>(R) TACACGATCAGCACCTTGG | 139 |
| AF150904.1 | Myosin regulatory light chain 2 (*mylpf*) | (F) CGCTGAGGAGATGACCAACC<br>(R) CTTCTCCGTGTGTGATGACGTAGC | 98 |
| XM_020089620.1 | Ornithine carbamoyltransferase (*otc*) | (F) ACAGGAAGTGGACAATCATGGG<br>(R) CAGCCTGACTATTACACCACG | 114 |
| XM_020106478.1 | PDZ and LIM domain protein 3 (*pdli3*) | (F) ACCAGGACAGTAAAAGCCCC<br>(R) TCCCATTCCCACACTTGTCG | 91 |
| XM_035157586.1 | Troponin I (*tnni1*) | (F) ATTGATGTTGTGGACGAGGAG<br>(R) CAGGGTCGGTTTCTTGAACTTA | 123 |
| EB038831.1 | Ubiquitin (*ubq*) | (F) AAATCCACCGTCTGAGGCG<br>(R) GAAGCAGTAGGTGAGGCAGC | 113 |

Journal of Experimental Biology • Supplementary information

**Table S2.** Number of differentially expressed genes (DEGs) and differentially abundant proteins (DAPs) in white skeletal muscle identified in response to temperature-induced growth manipulations in juvenile Pacific halibut.

| | Growth suppression (9°Cvs2°C) | | Growth stimulation (9°C vs 2°C→9°C) | |
|---|---|---|---|---|
| **Differentially expressed genes (DEGs)** | | | | |
| | **Total** | **Annotated** | **Total** | **Annotated** |
| All DEGs | 1619 | 1187 | 815 | 610 |
| Up-regulated | 716 | 511 | 282 | 202 |
| Down-regulated | 903 | 676 | 533 | 408 |
| | | | | |
| **Differentially abundant proteins (DAPs)** | | | | |
| | **Annotated** | | **Annotated** | |
| All DAPs | 241 | | 159 | |
| Increased abundance | 91 | | 149 | |
| Decreased abundance | 150 | | 10 | |

**Table S3.** List of down-regulated differentially expressed genes (DEGs) in white skeletal muscle in response to growth suppression by low-temperature acclimation (9ºC vs 2ºC).

Available for download at
https://journals.biologists.com/jeb/article-lookup/doi/10.1242/jeb.251013#supplementary-data

**Table S4.** Enrichment of Gene Ontology classes of down-regulated differentially expressed genes (DEGs) in white skeletal muscle identified in response to growth suppression by low-temperature acclimation (9ºC vs 2ºC).

Available for download at
https://journals.biologists.com/jeb/article-lookup/doi/10.1242/jeb.251013#supplementary-data

**Table S5**. List of up-regulated differentially expressed genes (DEGs) in white skeletal muscle in response to growth stimulation (9ºC vs 2ºC→9ºC).

Available for download at
https://journals.biologists.com/jeb/article-lookup/doi/10.1242/jeb.251013#supplementary-data

Journal of Experimental Biology • Supplementary information

**Table S6.** Enrichment of Gene Ontology classes of up-regulated differentially expressed genes (DEGs) in white skeletal muscle identified in response to growth stimulation (9ºC vs 2ºC→9ºC).

Available for download at
https://journals.biologists.com/jeb/article-lookup/doi/10.1242/jeb.251013#supplementary-data

**Table S7.** List of differentially abundant proteins (DAPs) in white skeletal muscle in response to growth suppression by low-temperature acclimation (9ºC vs 2ºC).

Available for download at
https://journals.biologists.com/jeb/article-lookup/doi/10.1242/jeb.251013#supplementary-data

**Table S8.** List of differentially abundant proteins (DAPs) in white skeletal muscle in response to growth stimulation (9ºC vs 2ºC→9ºC).

Available for download at
https://journals.biologists.com/jeb/article-lookup/doi/10.1242/jeb.251013#supplementary-data

**Table S9**. Enrichment of Gene Ontology classes of differentially abundant proteins (DAPs) in white skeletal muscle identified in response to growth suppression by low-temperature acclimation (9ºC vs 2ºC).

Available for download at
https://journals.biologists.com/jeb/article-lookup/doi/10.1242/jeb.251013#supplementary-data

**Table S10.** Enrichment of Gene Ontology classes of differentially abundant proteins (DAPs) in white skeletal muscle identified in response to growth stimulation (9ºC vs 2ºC→9ºC).

Available for download at https://journals.biologists.com/jeb/article-lookup/doi/10.1242/jeb.251013#supplementary-data

**Table S11.** Minimum, median and maximum Bayesian standard ellipse area estimates for each of the temperature treatments.

| | Bayesian Standard Ellipse Area | | |
|---|---|---|---|
| **Temperature treatment** | **Minimum** | **Mean** | **Maximum** |
| **2°C** | 0.11 | 0.32 | 1.19 |
| **9°C** | 0.04 | 0.10 | 0.33 |
| **2°C→9°C** | 0.03 | 0.07 | 0.30 |

**Table S12.** Proportion overlap, median angle and median distance among Bayesian standard ellipses. Overlap is shown as a proportion of non-overlapping area. Polar vectors median angle and median distance among ellipse centroids are shown.

| | Overlap, Median Angle (°), Median Distance (‰) | | |
|---|---|---|---|
| **Temperature treatment** | **2°C** | **9°C** | **2°C→9°C** |
| **2°C** | | | |
| **9°C** | 0.06, 1.71, 0.83 | | |
| **2°C→9°C** | 0.04, -0.70, 0.95 | 0.11, 0.20, 0.66 | |

**Table S13.** C:N ratios for each of the temperature treatments.

| **Temperature treatment** | **C:N ratio (mean ± standard deviation)** |
|---|---|
| **2°C** | 4.00 ± 0.38 |
| **9°C** | 4.00 ± 0.22 |
| **2°C→9°C** | 3.79 ± 0.12 |

Journal of Experimental Biology • Supplementary information
